# Adoption value of serum hematological inflammatory markers in early diagnosis of necrotising enterocolitis

**DOI:** 10.5937/jomb2502244L

**Published:** 2025-03-21

**Authors:** Ruogu Luo, Jing Zhang, Xiaolong Li, Fei Peng, Anpeng Zhang, Pengfei Zhang

**Affiliations:** 1 Northwest Women and Children's Hospital, Department of General Surgery, Xi'an, China

**Keywords:** neonatal necrotising enterocolitis, inflammatory markers, diagnostic value, AUC, correlation analysis, neonatalni nekrotizirajući enterokolitis, inflamatorni markeri, dijagnostička vrednost, AUC, korelaciona analiza

## Abstract

**Background:**

Early diagnosis and intervention of neonatal necrotising enterocolitis (NEC) are crucial for improving prognosis. It was to assess the diagnostic and staging value of high-sensitivity C-reactive protein (hs-CRP), white blood cell count (WBC), and interleukin (IL)-6 levels in NEC, and to explore their correlation with disease severity.

**Methods:**

This retrospective study analysed clinical data from 43 NEC patients in the neonatology department of Northwest Women and Children's Hospital, designated as the experimental group (EG), and concurrently selected 38 healthy newborns as the control group (CG). Serum hsCRP, WBC, and IL-6 were measured in both groups. Statistical analyses were performed using SPSS 27.0.

**Results:**

hs-CRP, WBC, and IL-6 in NEC patients greatly surpassed those in healthy newborns, and these markers were notably positively correlated with NEC staging (r=0.756, 0.234, 0.901, P<0.05). Combined detection of hs-CRP, WBC, and IL-6 in early NEC diagnosis yielded an AUC of 0.988, with a sensitivity of 93.02% and specificity of 97.37%, all superior to individual detections (P<0.05).

**Conclusions:**

hs-CRP, WBC, and IL-6 are essential in diagnosing and assessing NEC, particularly combined detection, which greatly improves early diagnostic accuracy. Future research should further investigate additional inflammatory markers to optimise diagnostic methods, providing a more comprehensive scientific basis for early clinical intervention and treatment.

## Abstract (sr)

**Uvod:**

Rana dijagnoza i intervencija kod neonatalnog nekrotizirajućeg enterokolitisa (NEC) su ključne za poboljšanje prognoze. Cilj je bio da se proceni dijagnostička i stadijumska vrednost visoko osetljivog C-reaktivnog proteina (hs-CRP), broja leukocita (WBC) i nivoa interleukina (IL)-6 kod NEC-a, kao i da se istraži njihova korelacija sa težinom bolesti.

**Metode:**

Ova retrospektivna studija je analizirala kliničke podatke 43 pacijenta sa NEC-om na neonatološkom odeljenju Northwest bolnice za žene i decu, koji su činili eksperimentalnu grupu (EG), dok je istovremeno odabrano 38 zdravih novorođenčadi kao kontrolna grupa (CG). U obe grupe su mereni nivoi serumskog hs-CRP, broja leukocita (WBC) i IL-6. Statističke analize su izvršene korišćenjem softvera SPSS 27.0.

**Rezultati:**

Nivoi hs-CRP, WBC i IL-6 kod pacijenata sa NECom su bili znatno viši u poređenju sa zdravim novorođenčadima, a ovi markeri su pokazali značajnu pozitivnu korelaciju sa stadijumima NEC-a (r=0,756, 0,234, 0,901, P<0,05). Kombinovano određivanje hs-CRP, WBC i IL-6 u ranoj dijagnozi NEC-a rezultiralo je AUC od 0,988, sa osetljivošću od 93,02% i specifičnošću od 97,37%, što je bilo superiornije u odnosu na pojedinačna određivanja (P<0,05).

**Zaključak:**

hs-CRP, WBC i IL-6 su ključni u dijagnostikovanju i proceni NEC-a, posebno kada se koriste u kombinovanom određivanju, koje značajno poboljšava tačnost rane dijagnoze. Buduća istraživanja bi trebalo dodatno da ispitaju dodatne inflamatorne markere kako bi se optimizovale dijagnostičke metode, pružajući sveobuhvatniju naučnu osnovu za ranu kliničiku intervenciju i lečenje.

## Introduction

Neonatal necrotising enterocolitis (NEC) is characterised by inflammation and necrosis of intestinal tissues; NEC can progress to intestinal perforation and systemic infection, representing a common critical illness in neonates [1] [2]. The exact aetiology of NEC remains incompletely understood, but it is believed to result from a multifactorial interplay. Potential factors include immature intestinal development in premature infants, rendering them vulnerable to damage, or incomplete development of intestinal barrier function, making them susceptible to bacterial invasion [3] [4]. Hypoxia or hypotension can also lead to inadequate intestinal blood flow, while formula feeding may increase the risk of NEC. Clinical manifestations of NEC are diverse, with common symptoms including abdominal distension, feeding intolerance (vomiting or diarrhoea), abdominal wall erythema or swelling, bloody stools, and systemic symptoms such as fever, tachypnea, and lethargy [5]. Hence, early diagnosis of NEC is crucial for improving prognosis; recognition and intervention can prevent disease progression, reduce complications, and improve survival rates. Timely treatment can prevent the extension of necrotic areas, thereby avoiding intestinal perforation and systemic infection. It also helps reduce the risk of intestinal resection, lowering the incidence of long-term complications such as short bowel syndrome and malabsorption [6].

Early diagnosis of NEC involves clinical assessment and auxiliary examinations. Clinical assessment involves observing newborns' feeding patterns, abdominal distension, bloody stools, and systemic symptoms. Blood tests detect inflammatory markers, while abdominal X-ray and ultrasound can reveal abnormalities like intestinal gas, pneumoperitoneum, and bowel dilation [7]. However, clinical assessment is subjective and dependent on healthcare providers' experience and observation skills, which may lead to misdiagnosis or missed diagnosis. Additionally, newborns may present with atypical symptoms such as abdominal distension and feeding intolerance, which can also occur in other neonatal conditions, making it challenging to differentiate [8] [9]. In addition, abdominal X-ray examinations involve radiation exposure, albeit at low doses, which still carries potential risks, especially for premature and fragile newborns. In contrast, blood tests are relatively simple, requiring only a small blood sample, without the need for complex equipment or technology. By concurrently measuring these inflammatory factors, clinicians can obtain more comprehensive and accurate information regarding the inflammatory response, thereby assisting in making better diagnostic and treatment decisions.

This work aimed to assess the diagnostic and staging value of hs-CRP, WBC, and IL-6 in neonatal NEC and to explore the correlation of these inflammatory markers with the severity of NEC. Additionally, it aimed to compare the effectiveness of individual versus combined detection of these inflammatory markers in early NEC diagnosis to provide scientific evidence for clinical treatment and early intervention.

## Materials and methods

This retrospective study analysed 43 cases of neonatal NEC referred to the neonatology department of Northwest Women and Children's Hospital from June 2021 to June 2023. NEC staging was conducted according to the Bell-NEC criteria [10]. Inclusion criteria were: (1) hospitalisation for more than 24 hours, (2) no prior treatment history, (3) ability of the infants to cooperate with treatment and complete clinical data available, and (4) diagnosis of NEC according to the criteria in *Practical Neonatology*. Exclusion criteria were: (1) congenital anomalies such as gastrointestinal malformations, (2) concurrent organ failure, and (3) treatment abandonment by caregivers. 

When suspected symptoms of NEC appeared in infants, 1.0~1.5 mL of venous blood sample was immediately drawn and centrifuged at 3,000 rpm for 5 minutes. 0.5 mL of supernatant was then transferred into EP tubes and stored at -80°C in a freezer. All stored samples were analysed in batches within three months. hs-CRP was measured using an automated biochemical analyser (SD-1, Chengdu Seamaty Technology Co., Ltd.), WBC was analysed using a flow cytometry system (BD FACSymphony A5, Shanghai Medi-X BioTech Co., Ltd.), and IL-6 was assessed using ELISA kits (Shanghai Enzyme-linked Biotechnology Co., Ltd.) with a Varioskan LUX automated enzyme-linked analyser (produced by Thermo Fisher Scientific). Criteria for a positive diagnosis of NEC included hs-CRP>10 mg/L, WBC >20×10^9^/L, or IL-6>50 pg/mL; positivity in any of these parameters was sufficient for NEC diagnosis.

Data processing and statistical analysis were implemented employing the SPSS 27.0 software package. Normally distributed continuous data, presented as mean ± standard deviation, were compared employing independent samples t-tests. Logarithmic transformation was applied to achieve normality for non-normally distributed continuous data, followed by independent samples t-tests for comparison. One-way analysis of variance (ANOVA) was employed to compare hs-CRP, WBC, and IL-6 levels among different stages of NEC patients. Count data were presented as frequencies or percentages. ROC curves and AUC analysed the diagnostic value of hs-CRP, WBC, IL-6, and their combination for NEC. *P*<0.05 implied statistically significant.

## Results

The case group included 25 boys and 18 girls, with gestational ages of 27~36 weeks (mean: 33.73±2.56 weeks). 11 cases were stage I, 19 were stage II, and 13 were stage III. Concurrently, 38 healthy newborns were selected as the control group during the same period, comprising 18 boys and 20 girls, with gestational ages of 28~36 weeks (mean: 32.84±3.19 weeks). Table 1 shows that the two groups have some differences in their birth characteristics, with the case group having lower birth weight, length, and head circumference and lower Apgar scores at 1 and 5 minutes (*P*<0.05). However, there are no significant differences between the groups in terms of maternal age, education level, family income, mode of delivery, multiple births, or gestational age at birth (*P*>0.05). 

**Table 1:** Demographic Characteristics of Study Participants.

** Variable **	** Case Group (n=43) **	** Control Group (n=38) **	** p-value **
** Birth weight (g) **	1342.1±342.1, 800–2200	1521.9±281.9, 1000–2400	0.12
** Birth length (cm) **	44.2±3.5, 38–50	46.1±2.9, 40–52	0.04
** Head circumference (cm) **	30.5±2.1, 26–34	31.9±1.9, 28–36	0.01
** Apgar score at 1 minute **	6.4±1.5, 3–9	8.2±1.1, 6–10	<0.01
** Apgar score at 5 minutes **	8.1±1.2, 5–10	9.1±0.9, 7–10	<0.01
** Maternal age (years) **	28.5±4.2, 20–38	29.8±3.9, 22–40	0.23
** Maternal education level **			0.45
‣ High school	15 (34.9%)	12 (31.6%)	
‣ College	18 (41.9%)	16 (42.1%)	
‣ University	10 (23.3%)	10 (26.3%)	
** Family income **			0.67
‣ Low	8 (18.6%)	6 (15.8%)	
‣ Middle	20 (46.5%)	18 (47.4%)	
‣ High	15 (34.9%)	14 (36.8%)	
** Mode of delivery **			0.21
‣ Vaginal	25 (58.1%)	22 (57.9%)	
‣ Cesarean section	18 (41.9%)	16 (42.1%)	
** Multiple births **			0.56
‣ Singleton	38 (88.4%)	34 (89.5%)	
‣ Twin	4 (9.3%)	3 (7.9%)	
‣ Triplet	1 (2.3%)	1 (2.6%)	
** Gestational age at birth **			0.14
‣ <28 weeks	2 (4.7%)	1 (2.6%)	
‣ 28-32 weeks	15 (34.9%)	12 (31.6%)	
‣ 33-36 weeks	20 (46.5%)	18 (47.4%)	
‣ ≥37 weeks	6 (14.0%)	7 (18.4%)	

hs-CRP, WBC, and IL-6 in case group (53.82±9.37 ng/mL, 12.18±4.19×10^9^/L, 1842.53±238.24 pg/mL) notably surpassed those in the control group (4.24±0.56 ng/mL, 4.72±2.57×10^9^/L, 12.34±4.62 pg/mL), with marked differences between groups (*P*< 0.05). Specific data are shown in Figure 1.

**Figure 1:**
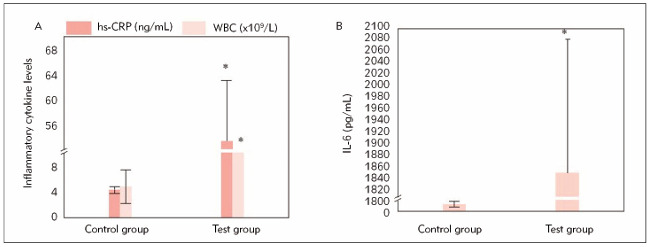
Comparison of hs-CRP, WBC, and IL-6 between groups. Note: A shows hs-CRP and WBC; B shows IL-6. * indicates marked differences relative to the control group (*P*<0.05).

hs-CRP, WBC, and IL-6 among patients at different stages of NEC showed drastic differences (*P*< 0.05) based on ANOVA. hs-CRP, WBC, and IL-6 were substantially higher in stage III versus stage II and stage I. Stage II showed notably higher levels than stage I. Specific data are detailed in Table 2.

**Table 2:** hs-CRP, WBC, and IL-6 levels in different stages. Note: * means that the difference was marked relative to stage I (*P*<0.05); # means that the difference was substantial compared with stage II (*P*< 0.05).

NEC staging	hs-CRP (ng/mL)	WBC (×10^9^/L)	IL-6 (pg/mL)
I (n=11)	38.25±9.45	9.53±3.06	15.67±8.56
II (n=19)	60.26±11.72*	12.88±4.35*	931.46±262.81*
III (n=13)	104.29±18.25*#	15.69±5.01*#	1836.24±278.46*#
F	76.046	6.150	182.131
P	<0.001	0.005	<0.001

hs-CRP, WBC, and IL-6 were positively correlated with staging (*r*=0.756, 0.234, 0.901, *P*<0.05). 

**Figure 2:**
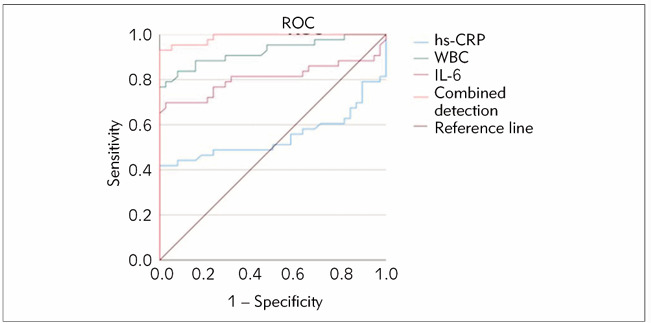
ROC curve of hs-CRP, WBC, and IL-6 diagnostic value.

For neonatal NEC, the combined detection of hs-CRP, WBC, and IL-6 showed higher values of AUC (0.988), sensitivity (93.02%), and specificity (97.37%) versus individual detection methods (*P*<0.05). Specific data are provided in Table 3, and ROC curves are illustrated in Figure 2.

**Table 3:** Diagnostic efficacy of hs-CRP, WBC, and IL-6.

Index	AUC	Standard error	Wald	95%CI	Sensitivity	Specificity
hs-CRP	0.549	0.353	7.059	(0.414,0.684)	69.77%(30/43)	73.68%(28/38)
WBC	0.925	0.319	0.909	(0.866,0.985)	83.72%(36/43)	89.47%(34/38)
IL-6	0.807	0.148	6.529	(0.703,0.912)	76.74%(33/43)	78.95%(30/38)
Combined<br>detection	0.988	5.656	10.473	(0.972,0.996)	93.02%(40/43)	97.37%(37/38)

## Discussion

Our study highlights the importance of hs-CRP, WBC, and IL-6 in diagnosing and assessing NEC. These biomarkers, particularly when combined, improve early diagnostic accuracy. Elevated levels of hs-CRP, WBC, and IL-6 are associated with NEC severity, making them useful tools for evaluating disease progression. Compared to other studies, our findings align with Elfarargy et al. [11], who found elevated hs-CRP levels in NEC patients, and Su et al. [12], who identified I-FABP, WBC, and PLT as prognostic indicators for NEC. Our study confirms the importance of hs-CRP in assessing disease severity and highlights IL-6 as a central player in the inflammatory response of NEC. The correlations between hs-CRP, WBC, and IL-6 levels and NEC staging were significant, with IL-6 showing the strongest positive correlation (r=0.901). This suggests that IL-6 is a reliable marker for assessing disease severity. While WBC has a weaker correlation, its combination with other markers provides valuable insights. Other studies, such as Huo et al. [13], also emphasise the importance of inflammatory biomarkers in NEC assessment. Pourcyrous et al. [14]. found that serial CRP measurements can aid in identifying infants with NEC stages II and III. At the same time, our study explores the diagnostic and staging value of hs-CRP, WBC, and IL-6 levels in NEC. The study by Mohd Amin et al. [15] investigated the predictive value of the C-reactive protein/albumin (CRP/ALB) ratio in predicting surgical intervention and mortality in neonates with NEC. Notably, both studies highlighted the importance of CRP levels in NEC management, but Mohd Amin et al. introduced a new aspect by combining CRP with albumin levels to create a ratio that can predict surgical intervention and mortality. Ibrohim et al. [16] investigated the association between prognostic factors and clinical deterioration in preterm neonates with NEC. Ibrohim et al. found that elevated CRP, along with late-onset of the disease, was an independent prognostic factor for clinical decline [16], as was our study. Morecroft et al. [17] investigated the role of plasma IL-6 and tumour necrosis factor (TNF) levels as predictors of disease severity and outcome in NEC.

## Conclusion

This study evaluated the diagnostic utility of hs-CRP, WBC, and IL-6 in NEC by comparing them between NEC patients and healthy neonates. Correlations between these inflammatory markers and NEC staging were explored alongside the diagnostic performance of single versus combined marker detection. Results indicated drastically elevated hs-CRP, WBC, and IL-6 in NEC patients versus healthy neonates, with a strong positive correlation observed with NEC staging. Combined detection of hs-CRP, WBC, and IL-6 showed considerably higher AUC values, sensitivity, and specificity in early NEC diagnosis relative to single marker detection, demonstrating superior diagnostic accuracy. However, this study focused exclusively on these three inflammatory markers, with potential limitations inherent in its retrospective design. Future research should expand sample sizes to validate further the applicability and reliability of combined marker detection across diverse populations. Additionally, exploring additional inflammatory biomarkers is warranted to comprehensively assess their roles in NEC diagnosis and prognosis, optimising and promoting multi-marker combined detection methods to enhance early diagnosis and treatment outcomes of NEC.

## Dodatak

### Conflict of interest statement

All the authors declare that they have no conflict of interest in this work.
